# Supplementary material for: Allosteric regulation and crystallographic fragment screening of SARS-CoV-2 NSP15 endoribonuclease
Source: Nucleic Acids Res. 2023 Apr 28;51(10):5255–70. doi: 10.1093/nar/gkad314 (PMC10250223; doi:10.1093/nar/gkad314)
Supplement: gkad314_Supplemental_Files [file gkad314_supplemental_files.zip › Supplementary datasets.zip!/159/Refine_4/01-BUSTER/Cycle-5/shell.01/R_Rfree.html]

# Rxpct factors in resolution bins at cycle 0


|  |  |  |  |  |  |  |  |  |
| --- | --- | --- | --- | --- | --- | --- | --- | --- |
|  | **<d> (Å)**  Rxpct\_Work | Rxpct\_Free | Rxpct\_All | |  |  |  |  |
|  | 12.8357 | 0.3381 | 0.4469 | 0.3427 |  |
|  | 7.4107 | 0.2802 | 0.2990 | 0.2813 |  |
|  | 5.7403 | 0.2511 | 0.2826 | 0.2524 |  |
|  | 4.8514 | 0.1908 | 0.2168 | 0.1922 |  |
|  | 4.2786 | 0.1849 | 0.1870 | 0.1850 |  |
|  | 3.8701 | 0.1940 | 0.2316 | 0.1958 |  |
|  | 3.5600 | 0.2077 | 0.2424 | 0.2094 |  |
|  | 3.3142 | 0.2100 | 0.2524 | 0.2126 |  |
|  | 3.1131 | 0.2061 | 0.2322 | 0.2073 |  |
|  | 2.9447 | 0.1984 | 0.2172 | 0.1992 |  |
|  | 2.8010 | 0.1923 | 0.2105 | 0.1932 |  |
|  | 2.6764 | 0.1891 | 0.2098 | 0.1902 |  |
|  | 2.5671 | 0.1876 | 0.1945 | 0.1880 |  |
|  | 2.4702 | 0.1861 | 0.1928 | 0.1864 |  |
|  | 2.3835 | 0.1863 | 0.2096 | 0.1874 |  |
|  | 2.3054 | 0.1780 | 0.2017 | 0.1791 |  |
|  | 2.2344 | 0.1928 | 0.2097 | 0.1936 |  |
|  | 2.1696 | 0.1957 | 0.2058 | 0.1962 |  |
|  | 2.1102 | 0.2078 | 0.2078 | 0.2078 |  |
|  | 2.0554 | 0.2354 | 0.2246 | 0.2348 |  |
|  | **Overall** | 0.2101 | 0.2348 | 0.2113 |  |

# Rxpct factors in resolution bins at cycle 1


|  |  |  |  |  |  |  |  |  |
| --- | --- | --- | --- | --- | --- | --- | --- | --- |
|  | **<d> (Å)**  Rxpct\_Work | Rxpct\_Free | Rxpct\_All | |  |  |  |  |
|  | 12.8357 | 0.3380 | 0.4468 | 0.3426 |  |
|  | 7.4107 | 0.2800 | 0.2988 | 0.2811 |  |
|  | 5.7403 | 0.2509 | 0.2826 | 0.2521 |  |
|  | 4.8514 | 0.1906 | 0.2167 | 0.1920 |  |
|  | 4.2786 | 0.1847 | 0.1869 | 0.1848 |  |
|  | 3.8701 | 0.1938 | 0.2315 | 0.1956 |  |
|  | 3.5600 | 0.2075 | 0.2423 | 0.2091 |  |
|  | 3.3142 | 0.2097 | 0.2522 | 0.2123 |  |
|  | 3.1131 | 0.2058 | 0.2321 | 0.2071 |  |
|  | 2.9447 | 0.1982 | 0.2174 | 0.1990 |  |
|  | 2.8010 | 0.1921 | 0.2105 | 0.1930 |  |
|  | 2.6764 | 0.1890 | 0.2098 | 0.1901 |  |
|  | 2.5671 | 0.1875 | 0.1947 | 0.1879 |  |
|  | 2.4702 | 0.1861 | 0.1928 | 0.1864 |  |
|  | 2.3835 | 0.1863 | 0.2095 | 0.1874 |  |
|  | 2.3054 | 0.1780 | 0.2018 | 0.1791 |  |
|  | 2.2344 | 0.1927 | 0.2096 | 0.1935 |  |
|  | 2.1696 | 0.1957 | 0.2059 | 0.1962 |  |
|  | 2.1102 | 0.2077 | 0.2078 | 0.2077 |  |
|  | 2.0554 | 0.2353 | 0.2248 | 0.2348 |  |
|  | **Overall** | 0.2099 | 0.2347 | 0.2112 |  |

# Rxpct factors in resolution bins at cycle 2


|  |  |  |  |  |  |  |  |  |
| --- | --- | --- | --- | --- | --- | --- | --- | --- |
|  | **<d> (Å)**  Rxpct\_Work | Rxpct\_Free | Rxpct\_All | |  |  |  |  |
|  | 12.8357 | 0.3377 | 0.4465 | 0.3423 |  |
|  | 7.4107 | 0.2796 | 0.2985 | 0.2807 |  |
|  | 5.7403 | 0.2506 | 0.2824 | 0.2518 |  |
|  | 4.8514 | 0.1903 | 0.2165 | 0.1918 |  |
|  | 4.2786 | 0.1846 | 0.1869 | 0.1847 |  |
|  | 3.8701 | 0.1937 | 0.2314 | 0.1955 |  |
|  | 3.5600 | 0.2073 | 0.2423 | 0.2090 |  |
|  | 3.3142 | 0.2096 | 0.2522 | 0.2121 |  |
|  | 3.1131 | 0.2057 | 0.2322 | 0.2069 |  |
|  | 2.9447 | 0.1981 | 0.2175 | 0.1990 |  |
|  | 2.8010 | 0.1920 | 0.2105 | 0.1929 |  |
|  | 2.6764 | 0.1889 | 0.2100 | 0.1901 |  |
|  | 2.5671 | 0.1874 | 0.1948 | 0.1879 |  |
|  | 2.4702 | 0.1861 | 0.1927 | 0.1865 |  |
|  | 2.3835 | 0.1864 | 0.2095 | 0.1874 |  |
|  | 2.3054 | 0.1780 | 0.2018 | 0.1791 |  |
|  | 2.2344 | 0.1927 | 0.2097 | 0.1935 |  |
|  | 2.1696 | 0.1957 | 0.2059 | 0.1962 |  |
|  | 2.1102 | 0.2077 | 0.2079 | 0.2078 |  |
|  | 2.0554 | 0.2354 | 0.2249 | 0.2349 |  |
|  | **Overall** | 0.2098 | 0.2347 | 0.2110 |  |

# Rxpct factors in resolution bins at cycle 3


|  |  |  |  |  |  |  |  |  |
| --- | --- | --- | --- | --- | --- | --- | --- | --- |
|  | **<d> (Å)**  Rxpct\_Work | Rxpct\_Free | Rxpct\_All | |  |  |  |  |
|  | 12.8357 | 0.3319 | 0.4435 | 0.3366 |  |
|  | 7.4107 | 0.2752 | 0.2951 | 0.2764 |  |
|  | 5.7403 | 0.2459 | 0.2784 | 0.2472 |  |
|  | 4.8514 | 0.1870 | 0.2150 | 0.1886 |  |
|  | 4.2786 | 0.1824 | 0.1869 | 0.1827 |  |
|  | 3.8701 | 0.1917 | 0.2327 | 0.1937 |  |
|  | 3.5600 | 0.2055 | 0.2401 | 0.2072 |  |
|  | 3.3142 | 0.2076 | 0.2502 | 0.2101 |  |
|  | 3.1131 | 0.2038 | 0.2330 | 0.2051 |  |
|  | 2.9447 | 0.1974 | 0.2199 | 0.1984 |  |
|  | 2.8010 | 0.1913 | 0.2095 | 0.1922 |  |
|  | 2.6764 | 0.1890 | 0.2108 | 0.1902 |  |
|  | 2.5671 | 0.1876 | 0.1978 | 0.1883 |  |
|  | 2.4702 | 0.1865 | 0.1926 | 0.1868 |  |
|  | 2.3835 | 0.1873 | 0.2100 | 0.1883 |  |
|  | 2.3054 | 0.1782 | 0.2046 | 0.1795 |  |
|  | 2.2344 | 0.1928 | 0.2111 | 0.1936 |  |
|  | 2.1696 | 0.1960 | 0.2068 | 0.1966 |  |
|  | 2.1102 | 0.2082 | 0.2089 | 0.2083 |  |
|  | 2.0554 | 0.2368 | 0.2263 | 0.2363 |  |
|  | **Overall** | 0.2079 | 0.2342 | 0.2092 |  |
